# Correction: *In situ* laser irradiation: the kinetics of the changes in the nonlinear/linear optical parameters of As_50_Se_40_Sb_10_ thin films for photonic applications

**DOI:** 10.1039/d3ra90098c

**Published:** 2023-10-09

**Authors:** D. Sahoo, P. Priyadarshini, R. Dandela, D. Alagarasan, R. Ganesan, S. Varadharajaperumal, R. Naik

**Affiliations:** a Department of Engineering and Material Physics, ICT-IOC Bhubaneswar 751013 India ramakanta.naik@gmail.com; b Department of Industrial & Engineering Chemistry, ICT-IOC Bhubaneswar 751013 India; c Department of Physics, Indian Institute of Science Bangalore 560012 India; d Centre for Nano Science and Engineering, Indian Institute of Science Bangalore 560012 India

## Abstract

Correction for ‘*In situ* laser irradiation: the kinetics of the changes in the nonlinear/linear optical parameters of As_50_Se_40_Sb_10_ thin films for photonic applications’ by D. Sahoo *et al.*, *RSC Adv.*, 2021, **11**, 16015–16025, https://doi.org/10.1039/D1RA02368C.

The authors regret the omission of an acknowledgement in the original article. This acknowledgement is given below.

Dr Ramakanta Nayak would like to acknowledge support from Meity, DST and MOE for the use of MNCF and NNCF facilities.

The Royal Society of Chemistry apologises for these errors and any consequent inconvenience to authors and readers.

## Supplementary Material

